# Dysmorphology in the Era of Genomic Diagnosis

**DOI:** 10.3390/jpm10010018

**Published:** 2020-03-17

**Authors:** Anna C. E. Hurst, Nathaniel H. Robin

**Affiliations:** 1 Department of Genetics, University of Alabama at Birmingham, Birmingham, AL 35243, USA; acehurst@uab.edu; 2 Department of Pediatrics University of Alabama at Birmingham, Birmingham, AL 35243, USA; 3 Department of Surgery/Otolaryngology, University of Alabama at Birmingham, Birmingham, AL 35243, USA

**Keywords:** dysmorphology, genetic testing, nextgen panels

## Abstract

Genetic and genomic testing technologies have expanded beyond levels of diagnostic capability that were unimaginable even a few years ago. While this has significantly benefited clinicians in their care of patients and families, it has also altered how geneticists evaluate patients. One immediate example is the role of the dysmorphologic physical exam in the patient evaluation. While some have suggested that it is no longer necessary, we argue that the dysmorphologic physical exam is still essential, albeit in a different role.

Genetic and genomic testing technologies are advancing diagnostic capability to levels previously unimaginable. While nearly every medical specialty experiences technological advances, the impact in clinical genetics is proving to be rapid and dramatic [1]. The role of a geneticist is shifting from evaluating a patient to determine what test to order to now ordering a test and then evaluating the results to determine if the genotype matches the patient’s phenotype. This change is recognized among medical geneticists, but the shifting role may not be apparent to pediatricians and other subspecialists who refer patients for assessment. However, it is an important distinction that may impact providers in all aspects of medicine. As sequencing becomes widely available, pediatricians may initially order broad-based genetic testing themselves, then refer to a geneticist for assistance with interpretation of results. 

Next-generation sequencing (NGS) is becoming quickly integrated into routine clinical practice, allowing for tremendous progress in the diagnosis of genetic conditions and the discovery of new disease-related genes. This is important to all healthcare providers, as genetic syndromes are common, and account for a disproportionate amount of healthcare expenditures [2]. Some may look at these new forms of genetic testing as merely the latest in a long line of technological advances, just as G-banded chromosome analysis was the preferred cytogenetic genetic test until it was replaced as a first-tier test by chromosomal microarrays [3]. Today, geneticists rarely begin an evaluation testing one gene at a time, as NGS multi-gene panels and exome and genome sequencing (ES/GS) allows for testing dozens to hundreds to thousands genes simultaneously.

As testing methodologies improve, there is increased likelihood of making an accurate diagnosis. Furthermore, it is increasingly common for descriptive, clinical diagnoses to be replaced by a molecular diagnosis. The immediate consequence of previous advancements in genetic testing was improved diagnostic capabilities, but now genomic testing has enhanced not just the diagnostic rate but fundamentally altered the practice of clinical genetics. This is best seen in the changing role of the quintessential geneticist’s tool, the dysmorphologic physical exam.

The dysmorphologic physical exam has long been an essential part of the practice of clinical genetics [4]. The clinical geneticist recognizes subtle morphologic differences, categorizes them, and reaches a genetic syndrome diagnosis. Until recently, the dysmorphologic exam was the primary way to recognize a genetic syndrome diagnosis. While many pediatricians can recognize common and obvious dysmorphic syndromes, few non-geneticists were trained or experienced enough to perform a structured dysmorphologic exam, distinguish minor anomalies, recognize their significance, and identify the unifying diagnosis. The process is time consuming and requires a particular skillset combining careful observation, pattern recognition, and recall. To most pediatricians, the dysmorphologic exam is a black box—in goes a set of symptoms (distinct facial appearance, structural birth defects, neurodevelopmental delay) and out comes a diagnosis with a name often unknown to everyone but the clinical geneticist. 

Historically, if the genetic alteration was known, genetic testing may be used to confirm the clinical suspicion. A negative test would prompt a re-evaluation, additional testing, or the patient still may have been labeled with a clinical diagnosis. While genetic testing was an important component, the clinical assessment was driven by the phenotype, which was determined through the initial dysmorphologic exam (Figure 1a—traditional paradigm) [5].

NGS technologies have irrevocably altered this paradigm. Now, when faced with a dysmorphic child, the detailed clinical assessment and dysmorphologic exam is no longer required to drive the evaluation process. The clinician can assess the general situation and order a test that covers all known syndromic possibilities. For example, when evaluating a child with disproportionate short stature an initial response may be to send “the skeletal dysplasia multigene panel,” or if a patient has scoliosis and a dilated aortic root, one may “order the aortopathy panel.” Of course, there is a role for the exam in test selection, but it is not as detailed as it once had to be when clinicians could only test one gene at a time. Now, we can allow the broad molecular test to be our first step at sorting out the correct diagnosis. Even though the detailed exam is not the key determinate of initial test selection, the real value of the phenotypic findings arises later.

It is tempting to think that one could order a broad panel or ES/GS testing with minimal clinical evaluation. However, relying on genetic testing technology instead of clinical assessment is flawed and reflects a poor understanding of the diagnostic process. For one reason, the causative genetic variant is not known for every genetic syndrome. For example, current NextGen testing will detect the genetic cause in under half of familial aortopathy [6]. Furthermore, a precise clinical phenotype is essential to interpreting the many variants identified [7]. Furthermore, as with any genetic test, pre- and post-test genetic counseling is still essential. What is different with ES/GS compared to other genetic tests is that the phenotyping data is utilized after the test is obtained in interpreting the test results [7]. At that point, the clinician must compare the genomic results to the patient’s phenotype, review what is known about the genes with identified sequence variants and compare this information to the patient’s phenotype to see if it “fits” (Figure 1b—new paradigm).

In this manner, the importance of the clinical data is as important as ever, just in a new orientation. The dysmorphologic exam informs the interpretation and analysis of genomic sequencing data by bridging a link between patients and the laboratory, providing accurate clinical information by using standardized morphological descriptions [8,9] and human phenotype ontology (HPO; http://human-phenotype-ontology.github.io/ [10]) terms to assist the laboratory in the filtering process. 

“Filtering” is the process by which the thousands of variants that are detected in each genomic analysis is assessed. Most variants are benign, but careful analysis is needed to distinguish benign from disease-related variants. Accurate phenotyping improves filtering, allowing the lab to focus further analysis on genes with the highest clinical relevance. Without phenotyping, genetic variant results can be overwhelming and even meaningless. We have adopted the saying, “It doesn’t matter what you see in the genome if you can’t see the patient.” In other words, care must be taken to avoid inappropriate assignment of causation, and appropriate phenotyping informs this process.

Medical geneticists now face a time of transition. ES/GS is prompting a period of disruptive transformation, and such times are always uncomfortable. It is likely that clinical geneticists’ identities will evolve, as they will no longer be viewed as the master “diagnostic detective,” making rare diagnoses by identifying unusual and rare minor findings and recognizing a pattern. Instead their role will evolve in to the expert phenotyper who understands the value in subtle facial and physical distinctions and the relationships between embryology, craniofacial development, and molecular genetic pathways, and is now able to correlate these with molecular variants.

While uncomfortable, times of change are also exciting, offering new challenges and new opportunities. One obvious challenge will be to ensure that every patient has access this new testing, as it is still expensive. Negotiating such issues is, unfortunately, becoming an important skill for the genetics professional. However, the unique clinical skills as a dysmorphologist will remain the most important role for the geneticist in the genomics era, albeit in a different role than in years past. In this role we will prevent diagnostic errors by resisting the urge to view molecular genetic test results as conclusive when clinical findings contradict the genotype. In this way, it is really no different than what physicians have always done—thinking critically about the intersection of clinical phenotype and laboratory findings and ensuring that the patient is not forgotten.

## Figures and Tables

**Figure 1:**
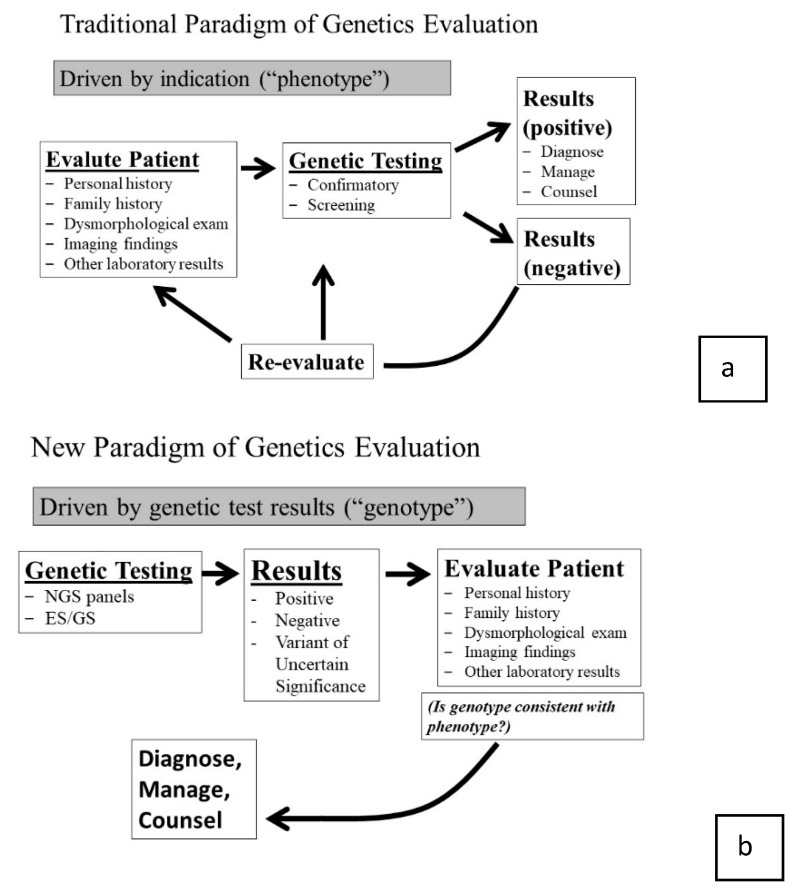
Comparing and contrasting the traditional (**a**) and new (**b**) paradigm of genetic evaluations. Traditionally, evaluation began as a phenotype-first approach. NGS has led to a genotype-first approach where the patient evaluation is more detailed after results have returned, and the geneticist must determine is the genotypic results are consistent with a diagnosis. (Adapted from NH Robin. [5]).

## Abbreviations

NGS	Next-Generation sequencing
ES	exome sequencing
GS	genome sequencing
